# The Role of Nutrition in Mitigating the Effects of COVID-19 from Infection through PASC

**DOI:** 10.3390/nu15040866

**Published:** 2023-02-08

**Authors:** Jacob T. Mey, John P. Kirwan, Christopher L. Axelrod

**Affiliations:** Pennington Biomedical Research Center, 6400 Perkins Road, Baton Rouge, LA 70808, USA

**Keywords:** COVID-19, PASC, long COVID, dietary patterns, nutrition, insulin resistance, immunity, inflammation

## Abstract

The expansive and rapid spread of the SARS-CoV-2 virus has resulted in a global pandemic of COVID-19 infection and disease. Though initially perceived to be acute in nature, many patients report persistent and recurrent symptoms beyond the infectious period. Emerging as a new epidemic, “long-COVID”, or post-acute sequelae of coronavirus disease (PASC), has substantially altered the lives of millions of people globally. Symptoms of both COVID-19 and PASC are individual, but share commonality to established respiratory viruses, which include but are not limited to chest pain, shortness of breath, fatigue, along with adverse metabolic and pulmonary health effects. Nutrition plays a critical role in immune function and metabolic health and thus is implicated in reducing risk or severity of symptoms for both COVID-19 and PASC. However, despite the impact of nutrition on these key physiological functions related to COVID-19 and PASC, the precise role of nutrition in COVID-19 infection and PASC onset or severity remains to be elucidated. This narrative review will discuss established and emerging nutrition approaches that may play a role in COVID-19 and PASC, with references to the established nutrition and clinical practice guidelines that should remain the primary resources for patients and practitioners.

## 1. Introduction & Background

Severe acute respiratory syndrome coronavirus 2 (SARS-CoV-2) is a novel coronavirus first identified in 2019. The virus quickly spread throughout the world, causing a global pandemic of respiratory illness, known now as COVID-19. As of late 2022, the COVID-19 pandemic has resulted in over 636 million confirmed cases and more than 6.6 million deaths worldwide [1]. COVID-19 causes severe acute respiratory syndrome, a potentially fatal condition, and spreads through contact with respiratory secretions such as saliva, mucus, or blood. A person can also contract the virus by inhaling droplets from an infected person while coughing or sneezing. The severity of COVID-19 symptoms vary from asymptomatic presentation [2], non-emergent symptoms such as mild fever, congestion, cough, and/or fatigue, and emergent symptoms such as difficulty breathing, chest pain, confusion, and lack of wakefulness necessitating hospitalization and critical care management. Many factors impact the severity of COVID-19 symptoms, including comorbidities, compromised immunity, advancing age, and even geographic location and air pollution [3].

Post-acute sequelae of COVID-19 (PASC) describes the condition of patients who continue to experience symptoms related to COVID-19 that persist for more than 3 months after initial infection [4]. The symptoms were initially documented in a case series of Italian patients, which found that nearly one-third of patients were still experiencing symptoms such as fatigue, muscle aches and pains, and cognitive difficulties 3 months post-infection [5]. While the long-term effects of COVID-19 are still not fully understood, PASC has emerged as a debilitating condition that requires both preventative and therapeutic approaches. More research is needed to better understand the causes and effects of PASC, and to develop effective prevention and treatment strategies. In the meantime, patients who are struggling with COVID-19 and PASC symptoms should seek medical help if they are having difficulty managing their symptoms. Current medical approaches to both COVID-19 and PASC are effective and undergoing continuous updates to improve patient outcomes.

Nutritional intervention, in combination with medical therapy, is a powerful tool to prevent and treat disease and disease-related symptoms. Emerging data from direct research on COVID-19 and PASC along with indirect research from similar respiratory virus infections and post-infection symptoms suggest potential roles for nutrition in clinical outcomes and symptoms management. Nutritional intake may play an indirect role on the risk of infection of COVID-19 through its effects on both innate and adaptive immunity. For example, being over- or under-nourished is related to more severe clinical outcomes and increased mortality in COVID-19 infection [6]. This holds true whether the over- or under-nutrition is chronic (i.e., high or low body weight, insulin resistance) or acute (i.e., hospitalized feeding). In addition, nutritional intake of key micronutrients and probiotics not only optimize immune function, it also plays a role in preventing viral infection and reducing the severity and length of viral infection symptoms. Finally, emerging nutritional approaches show positive effects on metabolic and pulmonary health, which may underlie COVID-19 and PASC pathology. This narrative review will cover emerging insights on the role of nutrition on COVID-19 and PASC in parallel with ongoing and established work related to viral infections along with metabolic and pulmonary health. In this way, we offer insight into the potential therapeutic impact of nutrition for both COVID-19 and PASC, from the risk for initial viral infection through the prolongation of symptoms.

## 2. Over/Undernutrition Effects on COVID-19 and PASC

### 2.1. Chronic Over/Undernutrition

Chronic over- or under-nourishment manifests in a BMI (Body Mass Index) of overweight (BMI ≥ 25 kg/m^2^)/obesity (BMI ≥ 30 kg/m^2^) or underweight (BMI ≤ 18.5 kg/m^2^), respectively. Both overweight and underweight BMI categories are associated with an increased risk of COVID-19/viral infections [7] and may exacerbate symptoms common to both acute COVID-19 infection and the chronic symptoms of PASC. In an analysis of nearly 150,000 U.S. adults infected with COVID-19, a nonlinear relationship with bodyweight and COVID-19 severity was revealed, whereby having a BMI in either the underweight or obesity categories increased risk, while having a BMI in the normal weight range or on the threshold between normal weight and overweight had the lowest risk [6]. Additionally, obesity *per se* has been linked to COVID-19 mortality and risk for intubation in multiple cohorts [8,9,10]. Tartof et al. assessed the relationship between obesity and COVID-19 related mortality in 6916 patients in southern California (US). Using a healthy BMI as a reference (18.5–24 kg/m^2^), individuals with a BMI between 40–44 kg/m^2^ had a relative risk of mortality of 2.68 and individuals with a BMI greater than 45 kg/m^2^ had relative risk of 4.18. Further, mortality risk with obesity was most apparent in males and individuals that were younger, while race, ethnicity, and other sociodemographic factors did not have an effect. Anderson et al. assessed the relationship between COVID-19 severity (using a composite of the clinical need for intubation and mortality) and obesity in 2466 adults in New York City, New York (US). Using individuals with an overweight BMI (25–30 kg/m^2^) as the reference, individuals with obesity had a greater risk of intubation or death, with the highest risk (hazard ratio of 1.6) in individuals with a BMI greater than 40 kg/m^2^; the hazard ratio was adjusted for age, sex, race/ethnicity, hypertension, asthma or chronic obstructive pulmonary disease, chronic kidney disease, pulmonary hypertension, smoking, cancer, and diabetes. A separate analysis was not presented for individuals with a BMI greater than 45 kg/m^2^. Similar to Tartof et al., this relationship between COVID-19 severity and obesity was more prominent in younger cohorts, i.e., age less than 65 years. In agreement with the Tartof and Anderson reports, Bennett et al., utilized data in the National COVID Cohort Collaborative and used machine learning to calculate the effect of obesity on COVID-19 severity while controlling for a large number of confounding variables. A total of 174,568 adults with COVID-19 were included and obesity was independently associated with higher clinical severity of COVID-19 (odds ratio 1.36). It is worthwhile to mention a conflicting report from a multi-center trial conducted across Brazil, which did not find a relationship between BMI and COVID-19 infection severity [11]. One explanation for this could be the relatively insensitive regression model used, which did not account for admission month, hospital urbanicity, insurance/payer type, or other previously identified confounding factors [12].

Multiple physiological mechanisms may explain the observed relationships between obesity and COVID-19 infection risk and severity. Chronic over-nutrition leads to obesity, which is independently associated with impaired metabolic function [13], pulmonary function [14], and inflammation [15]—risk factors for increased severity of disease. Obesity is also an independent risk factor for the diagnosis of PASC, as recently reported by the NIH RECOVER program [16]. Obesity also leads to impaired metabolic health, which has been reported to exacerbate both COVID-19 and PASC symptoms [17]. Though it is notable that in a recent systematic review, no relationship between overall metabolic health and risk of COVID-19 infection was observed. However, obesity *per se* and inflammation remained risk factors for COVID-19 infection and severity [7]. In another systematic review, COVID-19 infection and PASC were associated with increased risk of developing metabolic disease [18], suggesting a bi-directional relationship between COVID-19/PASC and metabolic health.

Lifestyle [19], pharmacological [20], and surgical [21] approaches are successful therapies to normalize nutritional intake and body weight, reducing the risk for impaired metabolic and pulmonary health and inflammation [22]. Given the profound positive effect of these weight-normalizing strategies on key risk factors related to COVID-19 and PASC, a prudent approach should involve improving bodyweight and chronic nutritional status. These successful approaches to weight management should be implemented with expert clinical supervision [23] and utilized in the context of an overall healthy dietary pattern as recommended by national dietary guidelines, such as the Dietary Guidelines for Americans [24], which broadly includes consuming a variety of fruits, vegetables, whole-grains, lean meats, fish, legumes, low-fat dairy, and nuts while limiting foods and beverages higher in added sugars, saturated fat, and sodium, and alcohol. In agreement, a systematic review in chronic pulmonary disease found that increasing fruit and vegetable intake improves pulmonary function with some evidence that it may improve systemic inflammation as well—potentially relevant to COVID-19 and PASC symptoms due to the effect on the pulmonary system [25].

In contrast to over-nutrition, chronic under-nutrition leads to malnutrition of not only insufficient calories (inadequate energy intake), but also key micronutrients that impact immune cell function. Expert consensus statements for individuals with malnutrition include both caloric and micronutrient provision, with a focus on vitamins A and D in malnourished patients with COVID-19 [26,27]. Given the availability of evidence, it is prudent to conclude that maintaining a healthy body weight and consuming a diet in accordance with national dietary guideline recommendations provides protection against COVID-19 and PASC onset and severity of symptoms.

### 2.2. A Critical Note on the Conversation Surrounding Obesity and COVID-19/PASC

The topic of chronic over-nutrition and COVID-19 requires careful consideration of the representation of persons with obesity by medical and lay communities. To this end, it is increasingly important to recognize that (1) obesity is a disease with clearly defined causative biological underpinnings and not a disorder of choice, laziness, or lack of personal motivation for self-care and (2) social stigmatization is harmful to individuals impacted by the disease and perpetuated by bias in medical care. To provide optimal patient care, researchers, clinicians, and the lay populous need to actively work together to combat the bias and stigma associated with obesity—minimally, by focusing on the use of person-first language along with other recommendations detailed in a recent joint international consensus statement [28] and patient-centered care report [29].

### 2.3. Acute over/undernutrition

While chronic over/undernutrition plays a role in COVID-19 and PASC risk, acute nutritional status also has an impact, specifically in severe symptoms of COVID-19 involving critical care or intensive care unit (ICU) admission. A literature review recently analyzed 10 clinical practice guidelines from across the globe to establish consensus practice recommendations for critically ill patients after COVID-19 infection [30]. Key nutrition concepts to optimizing patient care include assessing nutritional risk upon admission, estimating calorie needs (potentially through the utilization of indirect calorimetry), a slow and progressive provision of both energy and protein over the first 5–7 days of COVID-19 illness culminating in the provision of 20–30 kcal/kg/day with 1.2–2.0 g protein/day, and the use of oral or enteral nutrition within 48 h of admission. There was no agreement among the clinical practice guidelines regarding the use of low-carbohydrate formulas or omega-3 enriched formulas. It is noteworthy that over or underfeeding in critical care is associated with increased risk of morbidity and mortality [31,32,33]. This issue is complicated by the inherent difficulty calculating energy needs in COVID-19 patients, as during COVID-19 infection or even after resolution of infection, energy needs are variable and not well estimated by energy expenditure predictive equations [34]. Underfeeding has also been investigated in COVID-19-related admissions, where similar negative effects on health outcomes were observed [35]. It is critically important to improve our understanding of the changes in nutritional needs during COVID-19 and post-COVID-19 intensive care admission, as the timing of nutritional provision to align with patient needs has been established as a key factor in patient outcomes [36].

### 2.4. Dietary Factors That Affect Innate and Adaptive Immunity

The immune system is the body’s central defense network against infection and disease. The immunological responses to antigens or foreign molecules, such as viruses, involve the innate and adaptive immune cells [37]. Innate immunity is the body’s general first line of defense against pathogens. It includes physical barriers, such as the skin and mucous membranes, as well as chemical defenses, including acids and enzymes. Innate immunity also includes cells, such as natural killer cells, which can recognize and destroy pathogens. Adaptive immunity is the body’s second line of defense. It develops after exposure to a pathogen and confers long-lasting protection against that specific pathogen. Adaptive immunity is mediated primarily by B- and T-cells, which produce antibodies that bind to and neutralize pathogens. Innate and adaptive immunity are supported by dietary factors such as micronutrients and minerals. Adequate dietary intake of the micronutrients and minerals is achievable through consuming a healthy diet in accordance with national dietary guidelines [24]. However, supplementation may provide additional benefit to the immune system or combat viral infections and thus are implicated in COVID-19 and PASC therapy. Dietary factors of interest include vitamins C and D, zinc, iron, and probiotics and are discussed in further detail below.

### 2.5. Vitamin C

Ascorbic acid or vitamin C is a water-soluble vitamin and antioxidant that plays critical roles in both innate and adaptive immunity, including B and T-cell function, the extent to which has been reviewed previously [38]. Vitamin C deficiency, though increasingly rare in developed countries, impairs immune function and increases susceptibility to viral infection [39]. Supplementation of vitamin C protects against viral respiratory infection and even the common cold in individuals exposed to harsh environments (e.g., cold, intense exercise) or stressful conditions, with evidence remaining inconclusive under routine conditions [40,41]. Whether the positive effects in stressful conditions translate to COVID-19 or PASC is unclear and requires direct research. The available research on vitamin C for COVID-19 is largely limited to observational reports including findings from a retrospective chart review on patients treated in COVID-19 intensive care units [42] and self-reported supplement use in international populations [43], neither of which suggest a benefit. Further, the COVID A to Z multicenter randomized clinical trial showed no benefit to vitamin C, zinc, or a combination on the duration of COVID-19 symptoms in ambulatory patients [44]. Vitamin C supplementation in combination with COVID-19 therapy in critically ill patients did not lower mortality but did decrease the incidence of thrombosis [45]. Currently, there is insufficient evidence to support vitamin C supplementation for the prevention or treatment of COVID-19 or PASC [46].

### 2.6. Vitamin D

Vitamin D encompasses a class of fat-soluble secosteroids responsible for increasing calcium absorption and bone resorption. In addition, vitamin D also contributes to innate and adaptive immune function through production of metabolites and activation of vitamin D responsive elements. Although only present in a few foods naturally (e.g., fatty fish), vitamin D is fortified in foods and can be obtained from direct sunlight. Vitamin D status has been associated with COVID-19 infection and severity in some reports, but not others. However, subsequent meta-analyses and systematic reviews have revealed that both COVID-19 disease severity and mortality risk are increased with low vitamin D status [47]. Regarding vitamin D supplementation, a meta-analysis and systematic review of randomized controlled trials concludes a clear benefit for the prevention of viral respiratory infections [48]. Although there is a theoretical connection between data on viral respiratory infections and COVID-19, direct data on vitamin D supplementation from randomized clinical trials in COVID-19 is scarce and limits the ability to draw strong conclusions on the role of vitamin D in treating COVID-19. A systematic review and meta-analysis of primarily cohort studies, however, did conclude that there is a benefit for vitamin D supplementation in the reduction of COVID-19 severity, including mortality [49]. Despite this promising research, there is insufficient evidence to support vitamin D supplementation for the prevention or treatment of COVID-19 or PASC in healthy individuals [50], but there may be benefit for patients that present with diagnosed malnutrition, vitamin D deficiency [26,27], or poor vitamin D status [47].

### 2.7. Zinc

Zinc is a trace mineral obtained in the diet primarily through meat, fish, and other seafood along with fortified foods, like grain cereals [51]. Zinc is required for many enzymatic reactions related to innate and adaptive immunity, including antiviral defense mechanisms [52]. Accordingly, zinc deficiency results in a compromised immune system, with direct ramifications for immune cell function (B-cell, T-cell, and natural killer cell) [53]. Regarding COVID-19, serum zinc levels are lower in infected children [54] and adults [55] compared to non-infected individuals. Despite this research, zinc supplementation beyond recommended intakes has potential negative side effects such as causing copper deficiency and decreasing medication absorption [56]. Clinical trials using supplemental zinc to treat COVID-19 have been ineffective [44]. Thus, there is insufficient evidence to support zinc supplementation for the prevention or treatment of COVID-19 or PASC, and due to the potential negative long-term effects or interactions with medications, zinc supplementation is not recommended [57].

### 2.8. Vitamin C, D and Zinc Supplementation Summary

Taken together, maintaining a healthy diet with adequate vitamin C and D and zinc may support both innate and adaptive immunity to combat COVID-19, PASC, and related symptoms. Nutritional approaches to obtain C and D include consuming brightly colored fruits and vegetables (vitamin C), fortified milk and fatty fish (vitamin D), along with meats, other seafood, and fortified whole-grain cereals (zinc)—all parts of a healthy dietary pattern as described in national guideline recommendations. Supplementation of these nutrients in non-deficient individuals appears promising, but evidence is too limited currently to recommend supplementation as a preventative or therapeutic approach.

### 2.9. Iron

Iron is a mineral common in the diet and is obtained primarily through beef, chicken, fish, beans, and fortified grains, such as cereals. Iron is required for critical enzyme function of immune cells, such as neutrophils and lymphocytes [58]. As such, severe iron deficiency is associated with an increased risk of infection [59]. In contrast, excess iron levels also increase the risk of infection [60]. Notably, COVID-19 impacts iron homeostasis [61], and at least one report showed that iron levels were associated with COVID-19 severity [62]. In opposition to supplementation for COVID-19, some have instead proposed iron depletion therapy as a potential treatment strategy for COVID-19 due to its antiviral effect, although empirical data remains to be reported. Given the role of excess iron in infection risk and proposition for iron depletion therapy in COVID-19 treatment, iron supplementation for COVID-19 in otherwise healthy individuals is not warranted.

### 2.10. Probiotics and the Gut Microbiome

Apart from micronutrients and minerals, the gut microbiome plays an independent role in immune function. The gut microbiome consists of billions of living microorganisms, the composition of which is highly variable between individuals and responsive to diverse stimuli. These stimuli include, but are not limited to, the gastrointestinal environment, dietary patterns, pre-existing disease, genetics, and lifestyle factors. Probiotics are a mixture of living bacteria and/or yeast products that are obtained exogenously through the consumption of fermented foods, medications, or dietary supplements. Probiotics help to maintain floral diversity and gastrointestinal integrity. Probiotics improve immune function through multiple mechanisms and are directly impactful for reducing viral infections, including respiratory viruses [63,64]. Notably, emerging research suggests a connection between the gut microbiome and lung diseases [65], relevant to COVID-19 and PASC pathology. This two-way crosstalk includes altered gut microbiome (dysbiosis) in lung diseases and a potential therapeutic effect of resolving dysbiosis [66]. A recent systematic review incorporating 58 reports (49 preclinical studies, nine randomized clinical trials) concluded that manipulating the gut microbiota may benefit both innate and adaptive immunity [67]. Specific to COVID-19, probiotic supplementation in mild COVID-19 infection reduced fatigue [68]. Probiotic supplementation has also been tested as an adjuvant therapy to severe COVID-19 infection, reducing gastrointestinal and pulmonary symptoms along with reduced risk of transfer to an intensive care unit and mortality [69]. Further evidence regarding the importance of the gut microbiome is provided by a recent report that gut microbiome dysbiosis plays a causative role in COVID-19 severity [70]. In the case of PASC, a prospective cohort study analyzed fecal microbiome with shotgun metagenomics sequencing in 174 individuals with and without PASC and found that the composition of the gut microbiome was associated with the persistence of PASC [71]. Although it remains to be determined if nutritional alteration of the gut microbiome can be used in the prevention or treatment of PASC, a recent case report describes a 2-month nutrition intervention targeting the gut microbiome alleviated patient-specific PASC symptoms which included loss of appetite, nausea, anxiety, and heart palpitations [72].

### 2.11. Probiotics and the Gut Microbiome Summary

Taken together, maintaining a healthy gut microbiome may support both innate and adaptive immunity to combat COVID-19, PASC, and related symptoms. Nutritional approaches to support a healthy gut microbiome in humans includes eating a wide variety of foods across food groups [73] with a focus on fermented foods, such as yogurts and sauerkrauts [74]. The direct effect of probiotic supplementation on preventing COVID-19 and PASC onset or mitigating symptoms remains promising. Still, caution is required due to the limited evidence, which needs to be replicated in large, diverse populations for strong conclusions to be drawn.

## 3. Emerging Nutritional Approaches Relevant to COVID-19 and PASC

Impaired metabolic health is implicated in the increasing severity of both COVID-19 and PASC symptoms [17]. One emerging nutritional approach that may improve metabolic health is intermittent fasting [75]. Intermittent fasting does not dictate the amount or types of foods consumed, but rather establishes a stringent time frame for *when* food can be consumed. It is implicated in multiple metabolic pathways relevant to health and disease [76]. Intermittent fasting may also cause a spontaneous reduction in total caloric intake and help address issues with chronic over-nutrition/obesity. In these ways, through positive benefits on metabolic health and obesity, intermittent fasting is an intriguing nutritional approach with theoretical applications to both COVID-19 and PASC. Additional insight can be garnered from metabolic research in the pulmonary field, as reduced pulmonary function and respiratory failure are core components of severe COVID-19 and PASC symptoms. Recently asthma, which is a pulmonary disease that has many symptoms in common with COVID-19 and is also a comorbid condition related to elevated risk of COVID-19 and PASC severity, has been shown to have a metabolic underpinning [77]. The unique metabolic underpinning in asthma has yet to be fully elucidated but may involve a shift in fuel preference (i.e., nutrient utilization), away from carbohydrates towards lipids or ketones [78,79]. In agreement with the shift away from carbohydrate metabolism, ketosis shows a therapeutic benefit in murine asthma models by reducing bronchoconstriction in the airway smooth muscle; bronchoconstriction being the primary airway symptoms leading to wheezing and difficulty breathing [80]. A ketogenic diet formula has been investigated in clinical settings and has been shown to improve lung function by shifting nutrient utilization towards lipids and ketones [81]. Further, in more severe pulmonary diseases, a shift in fuel utilization towards lipids and ketones is associated with better clinical outcomes [82,83]. Given the importance of pulmonary health in COVID-19 and PASC pathology, ketogenic approaches represent a unique nutritional strategy that may theoretically combat COVID-19 and PASC. Notably, intermittent fasting increases the length of time and magnitude that ketones are used as fuel [84] and the combination of intermittent fasting-ketogenic protocols are used in medical practices that focus on low-carbohydrate approaches to improve metabolic health and body weight [85]. Despite these promising data, it is noteworthy that currently there is no direct evidence to support the use of intermittent fasting or ketogenic protocols for the prevention, treatment, and/or management of COVID-19 or PASC.

### A Brief Note on Current Medical Interventions for COVID-19 and PASC

Major limitations to drawing strong conclusions on novel nutrition therapies for COVID-19 and PASC are related to the paucity of data and discordant findings. These limitations are not unique to the nutrition field, as even pharmacological approaches in the prevention and treatment of COVID-19 and PASC are difficult to interpret currently. For example, compared to other diseases, the lack of large, placebo-controlled clinical trials hampers the evaluation of pharmacologic interventions for COVID-19 and PASC. Although many COVID-19 vaccine trials have been large and robust, COVID-19 medication trials are limited by relatively small sample sizes or a lack of randomization (e.g., open label), reducing the strength of the evidence. Despite these limitations, some patterns have emerged from the available data that are worth mentioning in a discussion surrounding potential therapeutic approaches for COVID-19 and PASC. Remdesivir, a broad-spectrum antiviral agent, reduces time to recovery from and severity of COVID-19 infection in hospitalized patients [86]. Dexamethazone, a glucocorticoid medication used to treat inflammatory reactions, reduced mortality in patients hospitalized with COVID-19, except for those receiving respiratory support [87]. In addition, the combination of baricitinib, a janus kinase inhibitor, and remdesivir produced the greatest clinical benefit related to time and severity of infection [88]. To this end, baricitinib plus remdesivir and dexamethasone plus remdesivir similarly improve survival in hospitalized patients with COVID-19, but dexamethasone increases the rate and severity of adverse events [89]. Other agents, such as hydroxychloroquine and chloroquine, are not effective in treating COVID-19 and may even be harmful [90]. PASC, though progressively diagnosable, lacks a clear biological and symptomatic presentation, making conceptualization and testing of pharmacotherapies difficult. A comprehensive overview of COVID-19/PASC pharmacologic treatments is beyond the scope of this review and interested readers are directed to the most recent clinical practice guidelines [91,92,93,94]. The importance of mentioning this is two-fold: (1) discussion about nutritional approaches should always been taken in the context of supporting established pharmacologic treatment models, not as a replacement for established clinical care approaches, and (2) it is critical to keep in mind that research on COVID-19 and PASC is limited due to the rapid and recent onset (c.a. 2020–2022) and was conducted in a volatile environment that included changes in viral prevalence, variations in the dominant viral strain, and constantly changing clinical treatment models. It can be anticipated that both pharmacological and nutritional approaches to COVID-19 and PASC will continue to evolve as larger, longer, and more rigorous studies are completed.

## 4. Additional Considerations: Nutrition across the Lifecycle

Nutrition recommendations for COVID-19 and PASC that apply to the adult population do not necessarily translate to other periods throughout the lifecycle. Even beginning prior to conception, nutrition needs of women of childbearing age are unique and impact both the mother and the fetus. Considerations continue beyond childbirth, where multiple factors, including breastfeeding status, affect nutritional needs of the mother and child. Detailed nutrition guidance is available through professional organizations, such as the American College of Obstetricians and Gynecologists (ACOG) [95] and the U.S. Department of Health and Human Services Office of Disease Prevention and Health Promotion, which publishes summary information relevant to pregnancy from multiple government sources, such as the Food and Drug Administration, U.S. Preventative Task Force, and the USDA’s Dietary Guidelines [96]. Other nutrition modifications may be required during the growth and adolescence of youth along with the other end of the lifecycle spectrum, advancing age and senescence [97]. Any nutrition modifications towards addressing COVID-19 and PASC should be made within the context of these individual lifecycle needs and in consultation with a primary care physician and dietitian.

## 5. Summary

**Figure 1:**
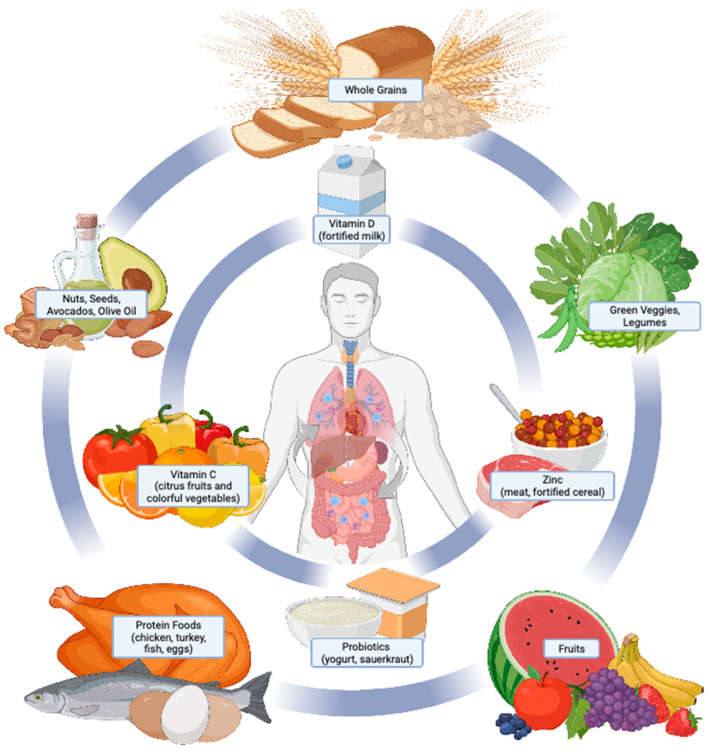
Overview of nutritional therapies in the treatment and management of COVID-19 and PASC. Nutrition plays a critical role in human health and the prevention and treatment of disease. Core body functions pertinent to COVID-19 and PASC onset and symptom severity include immune function, body weight regulation, metabolic health, and respiratory function. Effective nutritional strategies to support each of these functions include consuming a dietary pattern consisting of a variety of nutrient-dense foods (outer circle). Some foods provide essential vitamins, minerals, and probiotics, which are under investigation for their roles in COVID-19 and PASC (inner circle). Consuming foods from both the outer and inner circles as dietary staples aligns with a healthy dietary pattern as recommended by national dietary guidelines and the World Health Organization [98,99].

Numerous biological and environmental factors play a role in a person’s susceptibility to COVID-19 and PASC. Nutrition also plays a role in both COVID-19 infection and PASC, though the specific mechanisms are not yet fully understood (as shown in Figure 1). Individuals with chronic or acute over- and under-nutrition are at increased risk of contracting viral infections, and once infected, are at greater risk for developing more severe symptoms. In addition, many of the underlying health conditions that increase the risk for severe COVID-19/PASC symptoms, such as obesity, weakened immunity, and impaired metabolic health, are closely linked with suboptimal nutrition. Maintaining a healthy weight and a nutritious diet with adequate micronutrients, minerals and probiotics may potentially reduce COVID-19 and PASC symptoms. However, additional dietary supplementation beyond an adequate nutritional intake to combat COVID-19 or PASC is not currently supported. Emerging research on the effect of intermittent fasting or shifting nutrient utilization from carbohydrates towards lipids and ketones (e.g., ketogenic approaches) provide promising areas for future research. Importantly, given the paucity of data on the topic and volatile research environment, national dietary guidelines and professional practice guidelines should remain the key point of reference for both patients and practitioners.

## Data Availability

No data were generated as a part of this review.

## References

[1] (2020). WHO COVID-19 Dashboard. Geneva: World Health Organization. https://covid19.who.int/.

[2] Oran A.D.P., Topol E.J. (2020). Prevalence of Asymptomatic SARS-CoV-2 Infection. Ann. Intern. Med..

[3] Cormier S.A., Yamamoto A., Short K.R., Vu L., Suk W.A. (2022). Environmental Impacts on COVID-19: Mechanisms of Increased Susceptibility. Ann. Glob. Health.

[4] Ladds E., Rushforth A., Wieringa S., Taylor S., Rayner C., Husain L., Greenhalgh T. (2020). Persistent symptoms after Covid-19: Qualitative study of 114 “long Covid” patients and draft quality principles for services. BMC Health Serv. Res..

[5] Carfi A., Bernabei R., Landi F. (2020). Gemelli Against C-P-ACSG. Persistent Symptoms in Patients After Acute COVID-19. JAMA.

[6] Kompaniyets L., Goodman A.B., Belay B., Freedman D.S., Sucosky M.S., Lange S.J., Gundlapalli A.V., Boehmer T.K., Blanck H.M. (2021). Body Mass Index and Risk for COVID-19–Related Hospitalization, Intensive Care Unit Admission, Invasive Mechanical Ventilation, and Death—United States, March–December 2020. MMWR Morb. Mortal. Wkly Rep..

[7] Luo S., Liang Y., Wong T.H.T., Schooling C.M., Au Yeung S.L. (2022). Identifying factors contributing to increased susceptibility to COVID-19 risk: A systematic review of Mendelian randomization studies. Int. J. Epidemiol..

[8] Tartof S.Y., Qian L., Hong V., Wei R., Nadjafi R.F., Fischer H., Li Z., Shaw S.F., Caparosa S.L., Nau C.L. (2020). Obesity and Mortality Among Patients Diagnosed With COVID-19: Results From an Integrated Health Care Organization. Ann. Intern. Med..

[9] Anderson M.R., Ferrante A.W., Baldwin M.R. (2021). Body Mass Index and Risk for Intubation or Death in SARS-CoV-2 Infection. Ann. Intern. Med..

[10] Anderson M.R., Geleris J., Anderson D.R., Zucker J., Nobel Y.R., Freedberg D., Small-Saunders J., Rajagopalan K.N., Greendyk R., Chae S.R. (2020). Body Mass Index and Risk for Intubation or Death in SARS-CoV-2 Infection: A Retrospective Cohort Study. Ann. Intern. Med..

[11] Barros-Neto J.A., Mello C.S., Vasconcelos S.M.L., Badue G.S., Ferreira R.C., Andrade M.I.S., Nascimento C.Q.D., Macena M.L., Silva J.A.D., Clemente H.A. (2022). Association between being underweight and excess body weight before SARS coronavirus type 2 infection and clinical outcomes of coronavirus disease 2019: Multicenter study. Nutrition.

[12] Bennett T.D., Moffitt R.A., Hajagos J.G., Amor B., Anand A., Bissell M.M., Bradwell K.R., Bremer C., Byrd J.B., Denham A. (2021). Clinical Characterization and Prediction of Clinical Severity of SARS-CoV-2 Infection Among US Adults Using Data From the US National COVID Cohort Collaborative. JAMA Netw. Open..

[13] Hoddy K.K., Axelrod C.L., Mey J.T., Hari A., Beyl R.A., Blair J.B., Dantas W.S., Kirwan J.P. (2022). Insulin resistance persists despite a metabolically healthy obesity phenotype. Obesity (Silver Spring). Obesity.

[14] Molani Gol R., Rafraf M. (2021). Association between abdominal obesity and pulmonary function in apparently healthy adults: A systematic review. Obes. Res. Clin. Pract..

[15] Makki K., Froguel P., Wolowczuk I. (2013). Adipose tissue in obesity-related inflammation and insulin resistance: Cells, cytokines, and chemokines. ISRN Inflamm..

[16] Hill E., Mehta H., Sharma S., Mane K., Xie C., Cathey E., Loomba J., Russell S., Spratt H., DeWitt P.E. (2022). Risk Factors Associated with Post-Acute Sequelae of SARS-CoV-2 in an EHR Cohort: A National COVID Cohort Collaborative (N3C) Analysis as part of the NIH RECOVER program. medRxiv.

[17] Scherer P.E., Kirwan J.P., Rosen C.J. (2022). Post-acute sequelae of COVID-19: A metabolic perspective. Elife.

[18] Zhang T., Mei Q., Zhang Z., Walline J.H., Liu Y., Zhu H., Zhang S. (2022). Risk for newly diagnosed diabetes after COVID-19: A systematic review and meta-analysis. BMC Med..

[19] (2014). Look Ahead Research Group Eight-year weight losses with an intensive lifestyle intervention: The look AHEAD study. Obesity.

[20] Rudofsky G., Catarig A.M., Favre L., Grau K., Hafliger S., Thomann R., Schultes B. (2021). Real-world use of once-weekly semaglutide in patients with type 2 diabetes: Results from the SURE Switzerland multicentre, prospective, observational study. Diabetes Res. Clin. Pract..

[21] Kashyap S.R., Bhatt D.L., Wolski K., Watanabe R.M., Abdul-Ghani M., Abood B., Pothier C.E., Brethauer S., Nissen S., Gupta M. (2013). Metabolic effects of bariatric surgery in patients with moderate obesity and type 2 diabetes: Analysis of a randomized control trial comparing surgery with intensive medical treatment. Diabetes Care..

[22] Bianchi V.E. (2018). Weight loss is a critical factor to reduce inflammation. Clin. Nutr. ESPEN.

[23] Tewksbury C., Nwankwo R., Peterson J. (2022). Academy of Nutrition and Dietetics: Revised 2022 Standards of Practice and Standards of Professional Performance for Registered Dietitian Nutritionists (Competent, Proficient, and Expert) in Adult Weight Management. J. Acad. Nutr. Diet..

[24] U (2020). U.S. Department of Agriculture and U.S. Department of Health and Human Services. Dietary Guidelines for Americans, 2020–2025.

[25] Furulund E., Bemanian M., Berggren N., Madebo T., Rivedal S.H., Lid T.G., Fadnes L.T. (2021). Effects of Nutritional Interventions in Individuals with Chronic Obstructive Lung Disease: A Systematic Review of Randomized Controlled Trials. Int. J. Chron. Obstr. Pulm. Dis..

[26] Barazzoni R., Bischoff S.C., Busetto L., Cederholm T., Chourdakis M., Cuerda C., Delzenne N., Genton L., Schneider S., Singer P. (2021). Endorsed by the EC. Nutritional management of individuals with obesity and COVID-19: ESPEN expert statements and practical guidance. Clin. Nutr..

[27] Barazzoni R., Bischoff S.C., Breda J., Wickramasinghe K., Krznaric Z., Nitzan D., Pirlich M., Singer P. (2020). Endorsed by the EC. ESPEN expert statements and practical guidance for nutritional management of individuals with SARS-CoV-2 infection. Clin. Nutr..

[28] Rubino F., Puhl R.M., Cummings D.E., Eckel R.H., Ryan D.H., Mechanick J.I., Nadglowski J., Ramos Salas X., Schauer P.R., Twenefour D. (2020). Joint international consensus statement for ending stigma of obesity. Nat. Med..

[29] Cardel M.I., Newsome F.A., Pearl R.L., Ross K.M., Dillard J.R., Miller D.R., Hayes J.F., Wilfley D., Keel P.K., Dhurandhar E.J. (2022). Patient-Centered Care for Obesity: How Health Care Providers Can Treat Obesity While Actively Addressing Weight Stigma and Eating Disorder Risk. J. Acad. Nutr. Diet..

[30] Chapple L.S., Tatucu-Babet O.A., Lambell K.J., Fetterplace K., Ridley E.J. (2021). Nutrition guidelines for critically ill adults admitted with COVID-19: Is there consensus?. Clin. Nutr. ESPEN.

[31] Pradelli L., Adolph M., Calder P.C., Deutz N.E., Carmona T.G., Michael-Titus A.T., Muscaritoli M., Singer P. (2022). Commentary on “Guidelines for the provision of nutrition support therapy in the adult critically ill patient: The American Society for Parenteral and Enteral Nutrition”. JPEN J. Parenter Enteral. Nutr..

[32] Rice T.W., Bingham A.L., Braunschweig C., Compher C., McCall M., Patel J., McKeever L. (2022). Response to “Commentary on ‘Guidelines for the provision of nutrition support therapy in the adult critically ill patient: The American Society for Parenteral and Enteral Nutrition’”: Clarity, scientific rigor, and a call to action. JPEN J. Parenter Enteral. Nutr..

[33] Compher C., Bingham A.L., McCall M., Patel J., Rice T.W., Braunschweig C., McKeever L. (2022). Guidelines for the provision of nutrition support therapy in the adult critically ill patient: The American Society for Parenteral and Enteral Nutrition. JPEN J. Parenter Enteral. Nutr..

[34] von Renesse J., von Bonin S., Held H.C., Schneider R., Seifert A.M., Seifert L., Spieth P., Weitz J., Welsch T., Meisterfeld R. (2021). Energy requirements of long-term ventilated COVID-19 patients with resolved SARS-CoV-2 infection. Clin. Nutr. ESPEN.

[35] Liu G., Zhang S., Mao Z., Wang W., Hu H. (2020). Clinical significance of nutritional risk screening for older adult patients with COVID-19. Eur. J. Clin. Nutr..

[36] McKeever L., Peterson S.J., Lateef O., Braunschweig C. (2021). The Influence of Timing in Critical Care Nutrition. Annu. Rev. Nutr..

[37] Koeppen B.M. (2008). Berne & Levy Physiology.

[38] Cerullo G., Negro M., Parimbelli M., Pecoraro M., Perna S., Liguori G., Rondanelli M., Cena H., D’Antona G. (2020). The Long History of Vitamin C: From Prevention of the Common Cold to Potential Aid in the Treatment of COVID-19. Front Immunol..

[39] Carr A.C., Maggini S. (2017). Vitamin C and Immune Function. Nutrients.

[40] Douglas R.M., Hemila H., Chalker E., Treacy B. (2007). Vitamin C for preventing and treating the common cold. Cochrane Database Syst. Rev..

[41] Hemila H. (2004). Vitamin C supplementation and respiratory infections: A systematic review. Mil. Med..

[42] Krishnan S., Patel K., Desai R., Sule A., Paik P., Miller A., Barclay A., Cassella A., Lucaj J., Royster Y. (2020). Clinical comorbidities, characteristics, and outcomes of mechanically ventilated patients in the State of Michigan with SARS-CoV-2 pneumonia. J. Clin. Anesth..

[43] Louca P., Murray B., Klaser K., Graham M.S., Mazidi M., Leeming E.R., Thompson E., Bowyer R., Drew D.A., Nguyen L.H. (2021). Modest effects of dietary supplements during the COVID-19 pandemic: Insights from 445 850 users of the COVID-19 Symptom Study app. BMJ Nutr. Prev. Health.

[44] Thomas S., Patel D., Bittel B., Wolski K., Wang Q., Kumar A., Il’Giovine Z.J., Mehra R., McWilliams C., Nissen S.E. (2021). Effect of High-Dose Zinc and Ascorbic Acid Supplementation vs Usual Care on Symptom Length and Reduction Among Ambulatory Patients With SARS-CoV-2 Infection: The COVID A to Z Randomized Clinical Trial. JAMA Netw. Open..

[45] Al Sulaiman K., Aljuhani O., Saleh K.B., Badreldin H.A., Al Harthi A., Alenazi M., Alharbi A., Algarni R., Al Harbi S., Alhammad A.M. (2021). Ascorbic acid as an adjunctive therapy in critically ill patients with COVID-19: A propensity score matched study. Sci. Rep..

[46] National Institutes of Health (2021). COVID-19 Treatment Guidelines, Vitamin C. https://www.covid19treatmentguidelines.nih.gov/therapies/supplements/vitamin-c/.

[47] Nicoll R., Henein M.Y. (2022). COVID-19 Prevention: Vitamin D Is Still a Valid Remedy. J. Clin. Med..

[48] Jolliffe D.A., Camargo C.A., Sluyter J.D., Aglipay M., Aloia J.F., Ganmaa D., Bergman P., Bischoff-Ferrari H.A., Borzutzky A., Damsgaard C.T. (2021). Vitamin D supplementation to prevent acute respiratory infections: A systematic review and meta-analysis of aggregate data from randomised controlled trials. Lancet Diabetes Endocrinol..

[49] D’Ecclesiis O., Gavioli C., Martinoli C., Raimondi S., Chiocca S., Miccolo C., Bossi P., Cortinovis D., Chiaradonna F., Palorini R. (2022). Vitamin D and SARS-CoV2 infection, severity and mortality: A systematic review and meta-analysis. PLoS ONE.

[50] National Institutes of Health (2021). COVID-19 Treatment Guidelines, Vitamin D. https://www.covid19treatmentguidelines.nih.gov/therapies/supplements/vitamin-d/.

[51] Ross A.C., Caballero B.H., Cousins R.J., Tucker K.L., Ziegler T.R. (2012). Modern Nutrition in Health and Disease.

[52] Read S.A., Obeid S., Ahlenstiel C., Ahlenstiel G. (2019). The Role of Zinc in Antiviral Immunity. Adv. Nutr..

[53] Justiz Vaillant A.A., Qurie A. (2022). Immunodeficiency.

[54] Dogan A., Dumanoglu Dogan I., Uyanik M., Kole M.T., Pismisoglu K. (2022). The Clinical Significance of Vitamin D and Zinc Levels with Respect to Immune Response in COVID-19 Positive Children. J. Trop. Pediatr..

[55] Elham A.S., Azam K., Azam J., Mostafa L., Nasrin B., Marzieh N. (2021). Serum vitamin D, calcium, and zinc levels in patients with COVID-19. Clin. Nutr. ESPEN.

[56] Office of Dietary Supplements, National Institutes of Health (2022). Zinc Fact Sheet for Health Professionals. https://ods.od.nih.gov/factsheets/Zinc-HealthProfessional.

[57] National Institutes of Health (2022). COVID-19 Treatment Guidelines, Zinc. https://www.covid19treatmentguidelines.nih.gov/therapies/supplements/zinc/.

[58] Tom B. (1999). Nutritional Biochemistry.

[59] Kumar V., Choudhry V.P. (2010). Iron deficiency and infection. Indian J. Pediatr..

[60] Litton E., Lim J. (2019). Iron Metabolism: An Emerging Therapeutic Target in Critical Illness. Crit. Care.

[61] Sonnweber T., Boehm A., Sahanic S., Pizzini A., Aichner M., Sonnweber B., Kurz K., Koppelstätter S., Haschka D., Petzer V. (2020). Persisting alterations of iron homeostasis in COVID-19 are associated with non-resolving lung pathologies and poor patients’ performance: A prospective observational cohort study. Respir Res..

[62] Hippchen T., Altamura S., Muckenthaler M.U., Merle U. (2020). Hypoferremia is Associated With Increased Hospitalization and Oxygen Demand in COVID-19 Patients. Hemasphere.

[63] Hao Q., Dong B.R., Wu T. (2015). Probiotics for preventing acute upper respiratory tract infections. Cochrane Database Syst. Rev..

[64] Saeterdal I., Underland V., Nilsen E.S. (2012). The effect of probiotics for preventing acute upper respiratory tract infections. Glob. Adv. Health Med..

[65] Zhang D., Li S., Wang N., Tan H.Y., Zhang Z., Feng Y. (2020). The Cross-Talk Between Gut Microbiota and Lungs in Common Lung Diseases. Front. Microbiol..

[66] Budden K.F., Gellatly S.L., Wood D.L., Cooper M.A., Morrison M., Hugenholtz P., Hansbro P.M. (2017). Emerging pathogenic links between microbiota and the gut-lung axis. Nat. Rev. Microbiol..

[67] Shi H.Y., Zhu X., Li W.L., Mak J.W.Y., Wong S.H., Zhu S.T., Guo S.L., Chan F.K.L., Zhang S.T., Ng S.C. (2021). Modulation of gut microbiota protects against viral respiratory tract infections: A systematic review of animal and clinical studies. Eur. J. Nutr..

[68] Rathi A., Jadhav S.B., Shah N. (2021). A Randomized Controlled Trial of the Efficacy of Systemic Enzymes and Probiotics in the Resolution of Post-COVID Fatigue. Medicines.

[69] d’Ettorre G., Ceccarelli G., Marazzato M., Campagna G., Pinacchio C., Alessandri F., Ruberto F., Rossi G., Celani L., Scagnolari C. (2020). Challenges in the Management of SARS-CoV2 Infection: The Role of Oral Bacteriotherapy as Complementary Therapeutic Strategy to Avoid the Progression of COVID-19. Front. Med..

[70] Venzon M., Bernard-Raichon L., Klein J., Axelrad J.E., Zhang C., Hussey G.A., Sullivan A.P., Casanovas-Massana A., Noval M.G., Valero-Jimenez A.M. (2022). Gut microbiome dysbiosis during COVID-19 is associated with increased risk for bacteremia and microbial translocation. bioRxiv.

[71] Liu Q., Mak J.W.Y., Su Q., Yeoh Y.K., Lui G.C., Ng S.S.S., Zhang F., Li A.Y.L., Lu W., Hui D.S. (2022). Gut microbiota dynamics in a prospective cohort of patients with post-acute COVID-19 syndrome. Gut.

[72] Wang Y., Wu G., Zhao L., Wang W. (2022). Nutritional Modulation of Gut Microbiota Alleviates Severe Gastrointestinal Symptoms in a Patient with Post-Acute COVID-19 Syndrome. mBio.

[73] Heiman M.L., Greenway F.L. (2016). A healthy gastrointestinal microbiome is dependent on dietary diversity. Mol. Metab..

[74] Guerra L.S., Cevallos-Cevallos J.M., Weckx S., Ruales J. (2022). Traditional Fermented Foods from Ecuador: A Review with a Focus on Microbial Diversity. Foods.

[75] Varady K. (2021). Intermittent fasting is gaining interest fast. Nat. Rev. Mol. Cell Biol..

[76] de Cabo R., Mattson M.P. (2019). Effects of Intermittent Fasting on Health, Aging, and Disease. N. Engl. J. Med..

[77] Perez M.K., Piedimonte G. (2014). Metabolic asthma: Is there a link between obesity, diabetes, and asthma?. Immunol. Allergy Clin. N. Am..

[78] Georas S.N., Wright R.J., Ivanova A., Israel E., LaVange L.M., Akuthota P., Carr T.F., Denlinger L.C., Fajt M.L., Kumar R. (2022). The Precision Interventions for Severe and/or Exacerbation-Prone (PrecISE) Asthma Network: An overview of Network organization, procedures, and interventions. J. Allergy Clin. Immunol..

[79] Mey J.T., Matuska B., Peterson L., Wyszynski P., Koo M., Sharp J., Pennington E., McCarroll S., Micklewright S., Zhang P. (2021). Resting Energy Expenditure Is Elevated in Asthma. Nutrients.

[80] Mank M.M., Reed L.F., Walton C.J., Barup M.L.T., Ather J.L., Poynter M.E. (2022). Therapeutic ketosis decreases methacholine hyperresponsiveness in mouse models of inherent obese asthma. Am. J. Physiol. Lung Cell Mol. Physiol..

[81] al-Saady N.M., Blackmore C.M., Bennett E.D. (1989). High fat, low carbohydrate, enteral feeding lowers PaCO_2_ and reduces the period of ventilation in artificially ventilated patients. Intensive Care Med..

[82] Heresi G.A., Mey J.T., Bartholomew J.R., Haddadin I.S., Tonelli A.R., Dweik R.A., Kirwan J.P., Kalhan S.C. (2020). Plasma metabolomic profile in chronic thromboembolic pulmonary hypertension. Pulm. Circ..

[83] Mey J.T., Hari A., Axelrod C.L., Fealy C.E., Erickson M.L., Kirwan J.P., Dweik R.A., Heresi G.A. (2020). Lipids and ketones dominate metabolism at the expense of glucose control in pulmonary arterial hypertension: A hyperglycaemic clamp and metabolomics study. Eur. Respir. J..

[84] Halberg N., Henriksen M., Söderhamn N., Stallknecht B., Ploug T., Schjerling P., Dela F. (2005). Effect of intermittent fasting and refeeding on insulin action in healthy men. J. Appl. Physiol.

[85] Gavidia K., Kalayjian T. (2021). Treating Diabetes Utilizing a Low Carbohydrate Ketogenic Diet and Intermittent Fasting without Significant Weight Loss: A Case Report. Front. Nutr..

[86] Beigel J.H., Tomashek K.M., Dodd L.E., Mehta A.K., Zingman B.S., Kalil A.C., Hohmann E., Chu H.Y., Luetkemeyer A., Kline S. (2020). Remdesivir for the Treatment of Covid-19-Final Report. N. Engl. J. Med..

[87] Horby P., Lim W.S., Emberson J.R., Mafham M., Bell J.L., Linsell L., Staplin N., Brightling C., Ustianowski A., Elmahi E. (2021). Dexamethasone in Hospitalized Patients with Covid-19. N. Engl. J. Med..

[88] Kalil A.C., Patterson T.F., Mehta A.K., Tomashek K.M., Wolfe C.R., Ghazaryan V., Marconi V.C., Ruiz-Palacios G.M., Hsieh L., Kline S. (2021). Baricitinib plus Remdesivir for Hospitalized Adults with Covid-19. N. Engl. J. Med..

[89] Wolfe C.R., Tomashek K.M., Patterson T.F., Gomez C.A., Marconi V.C., Jain M.K., Yang O.O., Paules C.I., Palacios G.M.R., Grossberg R. (2022). Baricitinib versus dexamethasone for adults hospitalised with COVID-19 (ACTT-4): A randomised, double-blind, double placebo-controlled trial. Lancet Respir. Med..

[90] Food and Drug Administration (2020). FDA Cautions Against Use of Hydroxychloroquine or Chloroquine for COVID-19 Outside of the HOSPITAL setting or a Clinical Trial due to Risk of Heart Rhythm Problems.

[91] Writing C., Gluckman T.J., Bhave N.M., Allen L.A., Chung E.H., Spatz E.S., Ammirati E., Baggish A.L., Bozkurt B., Cornwell W.K. (2022). 2022 ACC Expert Consensus Decision Pathway on Cardiovascular Sequelae of COVID-19 in Adults: Myocarditis and Other Myocardial Involvement, Post-Acute Sequelae of SARS-CoV-2 Infection, and Return to Play: A Report of the American College of Cardiology Solution Set Oversight Committee. J. Am. Coll. Cardiol..

[92] Fraile Navarro D., McMullan B., Bowen A.C., National C.-C.E.T. (2022). Clinical care of children and adolescents with COVID-19: Recommendations from the National COVID-19 Clinical Evidence Taskforce. Med. J. Aust..

[93] Rosenblum H.G., Wallace M., Godfrey M., Roper L.E., Hall E., Fleming-Dutra K.E., Link-Gelles R., Pilishvili T., Williams J., Moulia D.L. (2022). Interim Recommendations from the Advisory Committee on Immunization Practices for the Use of Bivalent Booster Doses of COVID-19 Vaccines-United States, October 2022. MMWR Morb. Mortal. Wkly Rep..

[94] (2021). “Living Guidance for Clinical Management of COVID-19” World Health Organization. https://www.who.int/publications/i/item/WHO-2019-nCoV-clinical-2021-2.

[95] (2021). Your Pregnancy and Childbirth: Month to Month.

[96] Anderson-Villaluz D., Quam J., Office of Disease Prevention and Health Promotion (2022). Nutrition during Pregnancy to Support a Healthy Mom and Baby. https://health.gov/news/202202/nutrition-during-pregnancy-support-healthy-mom-and-baby.

[97] Brown J.E. (2005). Nutrition through the Life Cycle.

[98] (2018). Food-Based Dietary Guidelines. Food and Agriculture Organization of the United Nations.

[99] Healthy Diet World Health Organization. https://www.who.int/news-room/fact-sheets/detail/healthy-diet.LastupdatedApril29,2020.

